# Integrated Genome-wide association and hypothalamus eQTL studies indicate a link between the circadian rhythm-related gene *PER1* and coping behavior

**DOI:** 10.1038/srep16264

**Published:** 2015-11-05

**Authors:** Siriluck Ponsuksili, Manuela Zebunke, Eduard Murani, Nares Trakooljul, Joachim Krieter, Birger Puppe, Manfred Schwerin, Klaus Wimmers

**Affiliations:** 1 Leibniz Institute for Farm Animal Biology (FBN), Institute for Genome Biology, Wilhelm-Stahl-Allee 2, 18196 Dummerstorf, Germany; 2 Leibniz Institute for Farm Animal Biology (FBN), Institute for Behavioral Physiology, Wilhelm-Stahl-Allee 2, 18196 Dummerstorf, Germany; 3 Institute of Animal Breeding and Husbandry, Christian-Albrechts-University, Kiel, Germany

## Abstract

Animal personality and coping styles are basic concepts for evaluating animal welfare. Struggling response of piglets in so-called backtests early in life reflects their coping strategy. Behavioral reactions of piglets in backtests have a moderate heritability, but their genetic basis largely remains unknown. Here, latency, duration and frequency of struggling attempts during one-minute backtests were repeatedly recorded of piglets at days 5, 12, 19, and 26. A genome-wide association study for backtest traits revealed 465 significant SNPs (FDR ≤ 0.05) mostly located in QTL (quantitative trait locus) regions on chromosome 3, 5, 12 and 16. In order to capture genes in these regions, 37 transcripts with significant SNPs were selected for expressionQTL analysis in the hypothalamus. Eight genes (ASGR1, CPAMD8, CTC1, FBXO39, IL19, LOC100511790, RAD51B, UBOX5) had cis- and five (RANGRF, PER1, PDZRN3, SH2D4B, LONP2) had trans-expressionQTL. In particular, for PER1, with known physiological implications for maintenance of circadian rhythms, a role in coping behavior was evidenced by confirmed association in an independent population. For CTC1 a cis-expression QTL and the consistent relationship of gene polymorphism, mRNA expression level and backtest traits promoted its link to coping style. GWAS and eQTL analyses uncovered positional and functional gene candidates for coping behavior.

Consistent behavior of animals along lifetime and/or across situations is an expression of an individual reaction pattern, temperament or animal personality^1^. In addition to behavioral and evolutionary sciences these concepts of animal personality gains interest with regard to animal welfare and ethical animal husbandry. In fact, animal welfare concerns play a significant role in the design of modern pig production systems. Another idea in this context is ‘coping’, which comprises all behavioral and physiological reactions in response to challenging, aversive situation. Coping styles evolved to form general adaptive response patterns with the “proactive or active pattern” characterized by a fight-or-flight response, high levels of aggression, and territorial control, where as the “reactive or passive pattern” shows a conservation-withdrawal response, immobility, and low aggression^2^,^3^,^4^. Both patterns differ in their behavioral, physiological and immunological characteristics. Between coping style and cortisol levels, the major indicator of stress, inverse relationships were shown in human and animals studies^5^,^6^,^7^,^8^. The behavior patterns of pigs living in confinement can vary according to several factors, but heredity is known to be a key factor in predicting temperament^9^. Defining phenotypes according to molecular features would promote the knowledge of functional traits like behaviour in both human and animal research. Knowledge of the genetic variations and molecular mechanisms which affect behavior facilitate the use of genetic or genomic selection is an alternative approach to increase the adaptability and reduce aggressive behavior of animals^10^,^11^. Indicators of behavior and aggressiveness in pigs include the backtest^12^, open-field test^13^, human approach test^14^, and counting skin lesions (lesion scoring)^15^,^16^. The backtest is a well-established, standardized test that has primarily been studied in relation to piglet behavior and other physiologic characteristics^12^,^17^,^18^,^19^. The backtest generates a clear behavioral response and provides insight into the behavioral flexibility of piglets^20^. The genetic parameters of the backtest like heritability were reported to vary from 0.10 to 0.5 depending on the sample size or test conditions^21^,^22^,^23^.

Several genomic regions and gene variants associated with stress response, aggression, and depression have been reported in both humans and animal models^24^,^25^,^26^. Pigs share numerous physiologic and genomic similarities with humans and therefore provide a tractable model in which to study the genetic determination of behavioral, physiological, and metabolic traits^27^. In fact, behavioral quantitative trait loci (QTL) have been mapped to stress^10^, feeding behavior^28^, maternal behavior^29^, and behavioral indices under healthy and disease conditions^30^ in porcine models.

Variation of complex traits is largely due to polymorphisms affecting regulatory sequences rather than coding sequences. Expression-QTL (eQTL) analysis integrates gene expression levels and genome-wide genotyping information to find genetic variation association with change in gene expression. Estimation of eQTL involves the handling of transcript abundances as phenotypes in linkage or association studies. Knowledge of the position of analyzed genes and markers enable differentiating cis and trans eQTL, with the first indicating a regulatory variation in the gene whose transcript level is recorded itself, whereas trans eQTL suggest a polymorphism elsewhere in the genome affecting the expression of the target gene^31^. Moreover, signals revealed by genome wide association studies (GWAS) are often located in regions with high linkage disequilibrium (LD) harboring several genes. ExpressionQTL analysis in relevant tissue point to regulatory variation and provide additional evidence for the role of genes within LD regions for a particular trait of interest. We have previously shown that the integration of GWAS revealing (clusters of) trait associated markers (regions) with eQTL analyses of trait-dependent expressed genes facilitates highlighting of genes related to complex traits like muscle and meat properties^32^. The genetic variants associated with coping behaviors in pigs remain largely unstudied. Here, we describe the first GWAS with subsequent eQTL analysis conducted to identify genetic variants responsible for coping behavior in two independent herds of pig.

## Results

A total of 294 German Landrace piglets were genotyped with the Illumina PorcineSNP60 BeadChip consisting of 62,163 loci. After filtering, 48,909 SNPs were used for further analysis. Backtest parameters (latency, duration, frequency of struggling at four time points) and coping type (high-reactive (HR), indistinct (IR), low-reactive (LR)) were used for association study (Table 1). 465 SNPs were significantly associated with at least one of the backtest traits. We measured cortisol levels, and the correlation coefficients of coping behavior and cortisol levels ranged between |0.11–0.20| and indicated significant correlation (p < 0.05, N = 290). However, no significant association between coping groups and cortisol levels was found. HR and LR showed a tendency of higher cortisol levels than IR groups.

The backtest traits were highly correlated. Latency showed negative correlation with duration and positive with frequency of struggling at each time point (r_s_ = |0.70–0.82|; p < 0.0001). The correlation between each time point of latency, duration and frequency of struggling ranged between r_s_ = |0.37–0.57|, r_s_ = |0.51–0.66| and r_s_ = |0.47–0.66|, respectively, with p < 0.0001. The summation of total latency (tL), total duration (tD), and total frequency (tF) and coping type were correlated with each other with highly significant coefficients of correlation ranging between |0.80–0.88|.

### Genome-wide association analysis of first response latency

**Figure 1:**
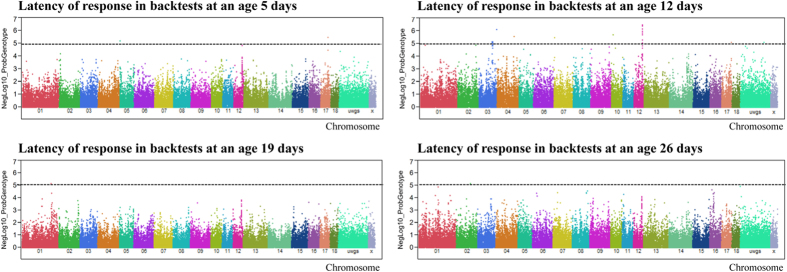
Genome-wide association with latency of response in backtests at ages of 5, 12, 19 and 26 days. Black lines at negative log10 (NegLog10) of 5 corresponds to FDR ≤ 0.05.

Manhattan plots of genome-wide association analyses of latency (L) at age 5 (Ld5), 12 (Ld12), 19 (Ld19), and 26 (Ld26) days are shown in Fig. 1. No SNPs were significantly associated with latency at days 5, 19, or 26 at a false discovery rate (FDR) of ≤5%, but 82 SNPs were significantly associated with Ld12, explaining about 7% of latency variance (Supplementary Table 1). Most of the significantly associated markers (N = 33, 40%) were located at 53–56 Mb of porcine chromosome (sus scrofa chromosome, SSC) 12. The most prominent gene located within this region is period circadian clock 1 (*PER1*). The top five markers significantly associated with Ld12 are shown in Table 2. Sixteen out of the 82 SNPs associated with Ld12 were located on SSC3 between 110 and 112 Mb.

### Genome-wide association analysis of response duration

**Figure 2:**
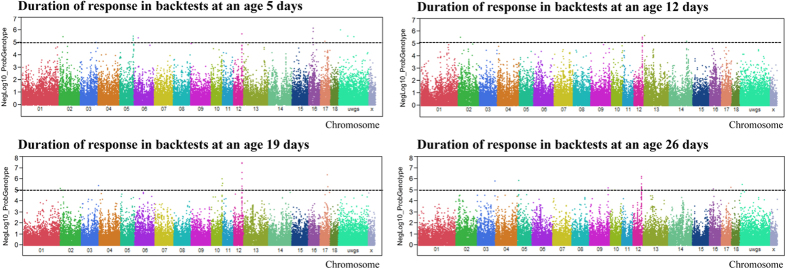
Genome-wide association with duration of response in backtests at ages of 5, 12, 19 and 26 days. Black lines at negative log10 (NegLog10) of 5 corresponds to FDR ≤ 0.05.

The top five markers significantly associated with Dd5, Dd12, Dd19 and Dd26 are shown in Table 2. Struggle duration at day 5 (Dd5) was significantly associated with 68 SNPs at FDR ≤ 5% (Fig. 2, Supplementary Table 2). These markers explained, on average, 7% of the phenotypic variance. The most prominent SNPs associated with Dd5 are located on SSC16 (31–32 Mb), SSC12 (55–56 Mb), and SSC5 (106–108 Mb). Two SNPs (ALGA0090009 and ALGA0090013) are located in a gene responsible for activating cyclic nucleotide-gated potassium channel 1 (*HCN1*) and are located on SSC16.

We found that Dd12 was significantly associated with 100 SNPs (FDR ≤ 5%) (Fig. 2, Supplementary Table 2). At SSC12 from 55–56 Mb there were 13 markers of interest and on SSC13 and 14 there were 12 and 10, respectively. Two SNPs (ALGA0090009 and ALGA0090013) on SSC16 were also found to be positively associated with struggle duration on Dd12.

Fifty-six SNPs were significantly (FDR ≤ 5%) associated with struggle duration at Dd19 (Fig. 2, Supplementary Table 2). Twenty-three of the 56 were located between 53 and 56 Mb of SSC12. Seven SNPs were located at 72–75 Mb on SSC10 and 4 SNPs were on SSC17.

Thirty-seven of the 101 SNPs associated with struggle duration on day 26 were found on SSC12 (Fig. 2, Supplementary Table 2). SNPs associated with Dd26 were also found on SSC 3, 5, 9, 13, 16, and 17.

### Genome-wide association analysis of struggle bout frequency

**Figure 3:**
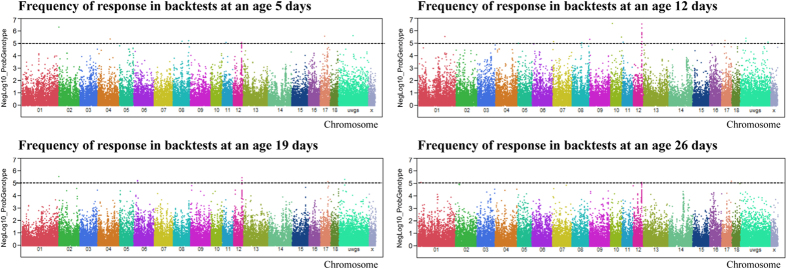
Genome-wide association with frequency of response in backtests at ages of 5, 12, 19 and 26 days. Black lines at negative log10 (NegLog10) of 5 corresponds to FDR ≤ 0.05.

Struggling bout frequency (F) at ages 5 (Fd5), 12 (Fd12), 19 (Fd19), and 26 (Fd26) days was significantly associated with 96, 124, 49, and 80 SNPs, respectively, at FDR ≤ 5% (Fig. 3, Supplementary Table 3). These markers explained, on average, 7% of the phenotypic variance. The top five markers significantly associated with Fd5, Fd12, Fd19 and Fd26 are shown in Table 2. Most of the SNPs associated with the frequency of struggling bouts at day 5 were found on SSC17, SSC12, SSC5, and SSC8. One SNP is located directly in the transcript of liprin-alpha-1-like (LOC100738060) on SSC2 with p < 1 × 10^−6^. This SNP was also significantly associated with the frequency of struggling bouts at days 12 and 19. At day 12, the frequency of struggling bouts was prominently associated with SNPs between 52 and 56 Mb on SSC12. ALGA0056876, on SSC10, was most strongly associated (p < 1 × 10^−6^) with Fd12. The markers associated with Fd19 were located on SSC12 (53–56 Mb), SSC6 (25 Mb), and SSC17 (50–56 Mb). The SNPs (ASGA0055092, ALGA0066975, ALGA0121951) located on SSC12 were within *PER1*. We also found that Fd26 was significantly associated with SNPs on SSC17 (37 and 64 Mb), SSC1 (22 Mb), and SSC12 (53–56 Mb).

### Genome-wide association analysis of total latency, duration, frequency, and coping type

**Figure 4:**
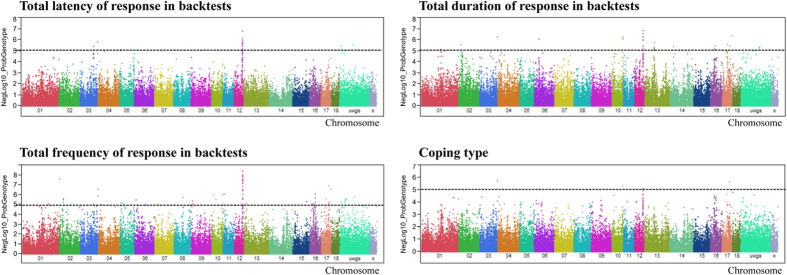
Genome-wide association with total latency, duration, frequency of response in backtests and backtest type. Black lines at negative log10 (NegLog10) of 5 corresponds to FDR ≤ 0.05.

**Figure 5:**
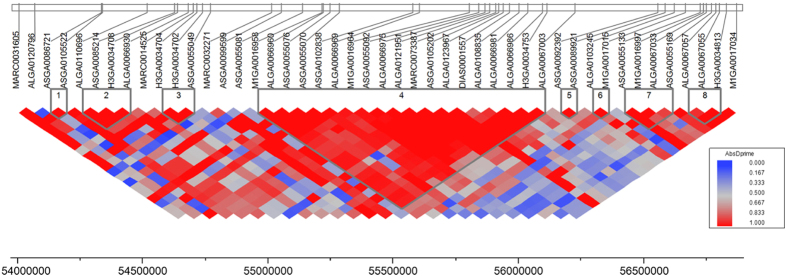
Linkage disequilibrium (LD) of single nucleotide polymorphisms (SNPs) located on SSC12 between 54 and 56 Mb that were significantly associated with backtest results. Color indicates the level of linkage disequilibrium between loci as depicted in the key. LD block 4 was the largest block located between 55 and 56 Mb.

The top ten markers significantly associated with total latency (tL), total response duration (tD), and total response frequency (tF) across testing at each age are shown in Table 3. Total latency, total duration, and total frequency were associated with 26, 40, and 69 SNPs, respectively, with p < 1 × 10^−5^ and FDR < 5% (Fig. 4, Supplementary Table 4). Eighteen of the 26 SNPs on SSC12 localized to *WRAP53*, *PER1*, *CTC1*, *RANGRF* and one on SSC3 localized to *ASGR1*. Twelve of the 40 SNPs associated with total duration localized to SSC12 and 9 of the 40 SNPs to SSC13. Finally, 22 of the 69 SNPs associated with total frequency localized to SSC12, with additional SNPs on SSC17 and SSC16. Together, the most prominent regions associated with total latency, total duration, and total frequency were located between 53 and 56 Mb on SSC12. The LD structure of SNPs located on SSC12 at 54–56 Mb is shown in Fig. 5. LD block 4 was the largest block identified, and was located between 55 and 56 Mb.

Ten SNPs were associated with coping type at FDR < 8%. Manhattan plots of genome-wide association analyses and the markers associated with coping type are shown in Fig. 4 and Table 3. These SNPs are located on SSC3, SSC6, SSC12, and some non-annotate in genome.

### Replication in second herd of crossbred piglets

Genotyping and association analysis of the most significant SNPs in *PER1* (ASGA0055092) and *CTC1* (ASGA0105202) in the first herd with 794 crossbred piglets from independent herds revealed significant association of ASGA0055092 in *PER1* with all backtest traits except Fd19; marker ASGA0105202 in *CTC1* was significantly associated with Fd12 only (Table 4).

### eQTL identification

**Figure 6:**
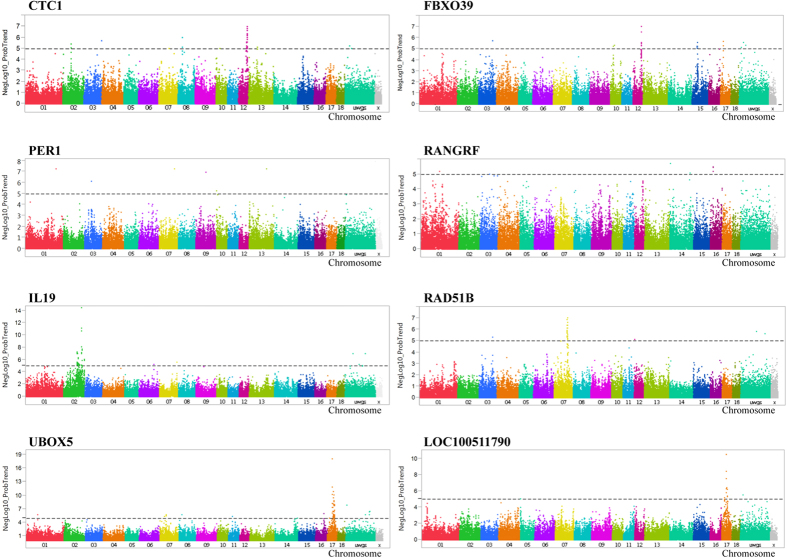
Genome-wide association for the abundance of candidate transcripts in porcine hypothalamus, which were significantly associated with backtest traits. Manhattan plots of genome-wide association analyses of transcript abundance of *CTC1*, *FBXO39*, *PER1*, *RANGRF*, *IL19*, *RAD51B*, *UBOX5* and *LOC10051790* are shown. Black lines at negative log10 (NegLog10) of 5 corresponds to FDR ≤ 0.05.

We detected eQTLs of 37 genes all containing SNPs significantly associated with backtest traits in the hypothalamus, an organ whose secretions are often responsible for behavioral traits (Supplementary Table 5). 326 eQTL (association of 304 SNPs with abundance of 13 transcripts) were identified at FDR ≤ 5% (Supplementary Table 6). Eight transcripts (*ASGR1*, *CPAMD8*, *CTC1*, *FBXO39*, *IL19, LOC100511790*, *RAD51B*, and *UBOX5*) contained cis eQTL (average p = 4 × 10^−7^). Another five transcripts (*RANGRF*, *PER1*, *PDZRN3*, *SH2D4B*, and *LONP2*) harbored trans eQTL (average p = 1.2 × 10^−6^). Representation of the cis and trans eQTL is shown in Fig. 6. In total, 38 out of the 465 SNPs that were significantly associated with the 13 backtest traits were also significantly associated with transcript levels of *CTC1, UBOX5, RAD51B, IL19, CPAMD, ASGR1* and *FBXO39*. Interestingly, all of them were cis eQTL.

## Discussion

The struggling response of piglets in a backtest early in life reflects the coping strategy and is a heritable and repeatable indicator of behavioral characteristics and of coping style also at later age. Some studies reported phenotypic and genetic correlation of backtests with other performance traits^17^,^23^. With a much larger data set of 3555 animals, of which the animals used here represent a subset, it was previously shown that the correlations among the backtest traits frequency, duration and latency of struggles were moderate to high at a single time point (r_s_ = |0.63–0.78|) and moderate between each trait at different time points (r_s_ = |0.19–0.44|); genetic correlations were high for all traits and time points (r_g_ > 0.8); this strongly supports the backtest traits as reliable parameters of personality and coping style^33^. Cortisol levels in plasma have been shown to be both directly and indirectly related to behavioral, physiological, and metabolic disease^15^,^34^. Our previous study detected a major quantitative trait locus at the position of the glucocorticoid receptor gene (NR3C1). SNP c.1829C>T, leading to a p.Ala610Val substitution in the ligand binding domain, showed a large effect on cortisol level and adrenal weight^35^. The genome-wide association study did not reveal any association of this QTL with coping style. Accordingly, we did not find significant association between cortisol levels and coping style in pigs as did Reimert *et al.*^19^. Backtest data were recorded early in life (<26 days) and cortisol level much later (180 days) in our study. The lack of association might be due to experimental reasons: the time gap may obscure a possible association between corticoid plasma concentrations and backtest traits. However, we have previously shown that the repeated backtests reflect personality and coping strategy with a moderate intra-individual consistency and heritability^33^, making the time gap less relevant and suggesting that basal cortisol levels and coping strategies are at best loosely functionally linked properties.

To our knowledge this is the first report of a genome-wide association study (GWAS) for backtest traits related to coping style in pigs. Most of the associated markers displayed peaks of closely linked loci that point to particular genomic regions. We identified 893 associations (465 SNPs) for 13 backtest traits at FDR ≤ 5%. The genomic region most frequently associated with backtest traits is located on SSC12 between 55 and 56 Mb, a region with high LD. Because of the large LD block, we decided to analyse eQTL effects of transcripts mapping to this regions in hypothalamus, which has implications for behavioral traits. The hypothalamus releases neurochemical signals to the paraventricular nucleus of the thalamus with in turn affects behavior^36^. Direct links between hypothalamic gene expression and aggressive behavior were shown in chicken^37^. Expression-QTL studies have been reported to detect cis-eQTL signals for more than 20% of catalogued GWAS hits for neurological disorders^38^, corroborating its relevance to our study. We identified candidate genes positioned in genomic regions with numerous trait-associated SNPs and with cis eQTL (*ASGR1*, *CPAMD8*, *CTC1*, *FBXO39*, *IL19*, *LOC100511790*, *RAD51B*, *UBOX5*) or trans eQTL (*RANGRF*, *PER1*, *PDZRN3*, *SH2D4B*, *LONP2*). Some SNPs associated with backtest traits are located in transcripts with plausible functions like *LOC100739126* (*NDEL1*), *WRAP53*, *C17H20orf26*, *HCN1*, *FCHSD2*, *SHQ1*, and *LOC100738060* (liprin-alpha-1-like) but were not associated with altered transcript levels.

The 6 markers associated with all 13 backtest traits were ASGA0055092, ASGA0105202, H3GA0034753, ALGA0066975, ALGA0121951, and MARC0073387, located on SSC12 (55–56 Mb) within *CTC1*, *PER1*, and *LOC100739126* (nuclear distribution protein nudE-like 1-like), i.e. genes with cis eQTL, trans-eQTL or plausible functions, respectively.

In fact, these SNPs were also associated with the transcript levels of *CTC1* in hypothalamus as cis eQTL. *CTC1* (CTS telomere maintenance complex component 1) encodes a component of the CST complex, which protects telomeres from degradation. Polymorphisms of CTC1 are causal for cerebroretinal microangiopathy^39^. The possible role of *CTC1* for behavior remains unclear, though there are indications of impact on the central and peripheral neurons. We found that the allele A of ASGA0105202 in *CTC1* is significantly more frequent in LR than HR; the allele is associated with higher expression of *CTC1*, and *CTC1* is higher expressed in in LR than HR. This consistent tri-angular relationship of polymorphism of the gene, variation of the phenotype and expression of the gene evidences *CTC1* as a gene contributing to genetic variation of coping behavior.

Indeed, eQTL complements the GWAS for backtest traits not only by pointing to the identical genomic regions but also to particular genes within these regions of high LD. ExpressionQTL, in particular cis eQTL, provides additional evidence for the association of the genes, as these genes harbor SNPs that are associated with the backtest traits and also with their own expression level, which is in turn associated with the backtest traits. Trait-associated SNPs are more likely to be eQTLs, in line with the role of regulatory SNPs in phenotypic variation. Consequently, the application of eQTL information can enhance discovery of trait-associated SNPs for complex phenotypes^38^,^40^.

The GWAS revealed an association between backtest traits and SNPs located in *PER1* with appealing implications for behavioral traits. Period circadian clock 1 (*PER1*) is a member of the period family genes and encodes components of circadian rhythm responsible for locomotion, metabolism, and behavior. The circadian rhythm pathway plays a key role in the maintenance of various endocrine, physiological, and behavioral functions. PER1-deficient mice were shown to display alterations in glucocorticoid rhythmicity^41^, innate routine behavior^42^ and feeding behavior^43^. Interestingly, a polymorphism in the human PER1 was associated with corticoid induced expression and mutant mice showed increased alcohol drinking in response to social defeat linking stress response and circadian rhythmicity - both being adaptations to exogenous factors^44^. In our study transcripts levels of *PER1* were regulated by a trans eQTL indicating that a yet to be identified non-regulatory polymorphism of *PER1* might be causative; while polymorphisms at the sites of the trans eQTL affect the expression of *PER1*. The SNP rs10720116 analyzed here is a synonymous coding transition obviously in linkage disequilibrium with the anticipated underlying SNP. The lack of association of cortisol levels and backtest traits in particular further suggests that a putative causal polymorphism does not affect the glucocorticoid induction of *PER1*. The association of *PER1* with backtest traits was shown here in two independent groups of animals, strongly promoting PER1 as a gene playing a significant role in coping behavior and linking coping behavior and a circadian rhythm-related gene shown to be involved in response to exogenous stimuli. Notable, no other circadian rhythm-related genes or SNP close to were detected by this GWAS.

The genes associated with backtest traits are related to neurogenesis and neuronal activity. For example, the nuclear distribution factor E-like gene regulates developmental neurogenesis and cortical neuronal positioning^45^,^46^. Genetic analyses revealed an association of nuclear distribution factor E (*NDE1*, or *NudE*) and its ortholog NDE-like 1 (*NDEL1*, or *Nudel*) with mental disorders^47^,^48^.

*WRAP53* is located on SSC12 (55–56 Mb), a region associated with backtest traits, and play important roles in telomere functions affecting the cell cycle^49^,^50^. *WRAP53* (WD repeat containing, antisense to TP53) has implications for the survival of motor neurons^51^. It encodes an essential component of the telomerase holoenzyme complex, is involved in the construction and maintenance of Cajal bodies, sub-organelles mediating RNA modifications.

Hyperpolarization-activated and cyclic nucleotide-gated (HCN) channels are encoded by four genes (*HCN1-4*) and are widely expressed throughout the heart and central nervous system. HCN1 is the predominant isoform known to be expressed in the hippocampus, neocortex, and cerebellar cortex^52^. HCN channels are involved in neuron excitation and synaptic activity in individual neurons and neuronal networks^53^. It has been reported that HCN1 channels constrain learning and memory and are involved in many diseases such as epilepsy, Alzheimer’s disease, and peripheral neuralgia^54^,^55^,^56^. Similarly, Liprin-α proteins are major protein constituents of synapses and contribute to synaptic transmission in different regions of the brain^57^,^58^. Knockdown of Liprin-α was reported to lead to a defect in presynaptic development and function^59^. The SNPs located in both HCN and Liprin-α were associated with coping style in this study but not with their transcript levels.

Other genes harboring cis eQTL, such as *UBOX5* and *FBXO39*, encode U box and F box protein and act as protein-ubiquitin ligases^60^,^61^. *RAD51* family members function in both mitotic and meiotic homologous recombination and DNA repair. *RAD51B* has been shown to be associated with breast cancer and Werner syndrome, which features progressive neurodegeneration^62^,^63^. The SNPs located in these 3 genes associated with coping behavior and also with their transcripts levels as cis eQTL.

An additional region of interest was SSC3 (139.8–139.9 Mb), with 3 SNPs (ALGA0107834/ASGR1, ALGA0066945, and ALGA0066946) associated with 8–13 backtest traits. The transcript levels of *ASGR1* were also associated with ALGA0066945 and ALGA0066946. *ASGR1* encodes a subunit of the asialoglycoprotein receptor and mediates the capture and endocytosis of galactose- (Gal) and N-acetylgalactosamine- (GalNAc) terminating glycoproteins. ASGR1 is highly expressed in hepatocytes^64^ and much weaker in other tissues including thyroid^65^ and peripheral blood monocytes^66^. Asialoglycoprotein receptors are involved in thyroglobulin transport, providing a link to thyroid gland hormones with implications for behavior^67^. We found that *ASGR1* is expressed in the hypothalamus and demonstrated that its transcripts levels are associated with SNP markers, which are also associated with coping style in pigs.

It was recently suggested that an imbalance of the immune system plays an important role in psychological stress and behavior^37^,^68^. SNPs located within the *CPAMD8* and *IL9* genes, both related to immune function, were associated with coping behavior and transcript levels (cis eQTL) in this study. *CPAMD8* is a member of the complement 3/alpha(2)-macroglobulin (C3/alpha(2)M) family and has functions in innate immunity^69^. It has been demonstrated that psychological stress enhances the production of immunosuppressive cytokines including IL19 via activation of beta-adrenoreceptors, which may impact stress-related disease processes^68^. Recently, it was demonstrated that the putative cis-acting haplotype of the *IL19* gene is associated with schizophrenia^70^. Together, the SNPs located in the *CPAMD8* and *IL9* genes were found to be associated with coping style, suggesting a relationship between the immune system and behavior.

Our results represent the first genome-wide SNP-based association analysis for behavioral traits related to coping type. Hypothalamic genes were identified within QTL regions showing considerable linkage disequilibrium using eQTL. The most highly associated region was SSC12 (53–56 Mb) harboring cis eQTL signals (*CTC1* and *FBXO39*) and trans eQTL signals (*PER1* and *RANGRF*). Other cis eQTL signals included *ASGR1* located on SSC3, *IL19* and *CPAMD8* located on SSC2, and *UBOX5* and *LOC10051179* located on SSC17. SNPs located on *HCN1* (SSC16) and *Liprin-α like* (SSC2), which have been linked to neuropathological disorders, showed no cis or trans eQTL signals. The genes identified in this study are known to be involved in telomere function signaling, immune function, ubiquitin system as well as the circadian rhythm pathway and neurotransmitter receptors, i.e. central nervous affairs related to behavior, offering perspectives to derive markers for breeding prediction. In particular, for *CTC1* and *PER1* with known physiological implications in the central nervous system, their role in behavior was evidenced by cis-eQTL and confirmed association in an independent population.

## Methods

### Ethics statement

All experiments were performed in accordance with relevant regulations and guidelines. Animals were provided by the Leibniz Institute for Farm Animal Biology (FBN). Animal care and tissue collection procedures followed the guidelines of the German Law of Animal Protection, and the experimental protocol was approved by the Animal Care Committee of the Leibniz Institute for Farm Animal Biology and the State Mecklenburg-Western Pomerania (Landesamt für Landwirtschaft, Lebensmittelsicherheit und Fischerei; LALLF M-V/TSD/7221.3-2.1-020/09). The experimental protocol was carried out in accordance with the approved guidelines for safeguarding good scientific practice at the institutions in the Leibniz Association.

### Animals, sample collection and trait measurement

German Landrace (DL) piglets (N = 294) were subjected to a backtest as previously described^33^. Briefly, we repeatedly performed a 1-min-backtest in domestic piglets at the ages of 5, 12, 19, and 26 days. Animals were put on their backs in a special V-shaped device. The test lasted for exactly 60 s and started as soon as the piglet was lying immobile in a supine position. The latency (L, time to first struggle), duration (D, cumulative time of struggling within the 1-min testing period), and frequency (F) of struggling bouts were recorded. The results from each parameter at each age tested were added together to reflect a summation of total latency (tL), total duration (tD), and total frequency (tF) for each individual piglet. Piglets were classified as high (HR), indistinct (IR), or low (LR) in reactivity, according to the following criteria: the latency of HR was ≤5 s and LR ≥ 35 s, the duration of HR was ≥25 s and LR ≤ 5 s, and the frequency of HR was ≥4 and LR ≤ 1^71^.

### Cortisol measurement

Total cortisol secretion was measured in the morning via the collection of 50 mL trunk blood from each pig during exsanguinations at about 180 days of age. Blood was collected in a plastic tube containing 1 mL of 0.5 M EDTA, stored on ice until plasma isolation, then stored long-term at −80 °C. Total plasma cortisol levels were determined using an enzyme-linked immunosorbent assay (DRG, Marburg, Germany) in duplicate according to the manufacturer’s protocol. The intra- and inter-assay coefficients of variation were ≤7.0% and 9.8%, respectively. Spearman’s rank correlation coefficient (Spearman’s rho, r_s_) and association between cortisol levels and coping behavior were also calculated.

### SNP genotyping

Genotyping was performed using the PorcineSNP60 BeadChip (Illumina Inc., San Diego, CA, USA) per manufacturer’s SNP Infinium HD assay protocol. In brief, 200 ng of DNA were amplified, fragmented, and hybridized to the PorcineSNP60 BeadChip containing 62,163 locus-specific 50-mers. Intensity data were measured and quality scores and genotypes were derived using the GenomeStudio software (Illumina Inc.). Call rates (<99%), minor-allele frequency (<5%), and deviation from Hardy-Weinberg equilibrium (p < 0.0001) were considered to fix the final set of markers for GWAS. The average call rate for all samples was 99.8 ± 0.2. The markers of the 60 K chip were mapped to the porcine reference genome, Sscrofa 10.2 (http://www.ensembl.org/).

### Genome wide association of coping behavior

The backtest parameters, latency and duration data were not normally distributed. These traits were converted into classes enabling Poisson distribution analysis. According to latency and duration, animals were assigned to four L-classes: class 1: 1–10 s; class 2: 11–20 s; class 3: 21–30 s; class 4: 31–60 s and five D-classes: class 1: 0 s; class 2: 1–10 s; class 3: 11–20 s; class 4:21–30 s; class 5: 31–60 s.

All backtest traits (latency, duration, and frequency) at ages 5, 12, 19, and 26 days, as well as the classification of piglets (coping type) based on backtest data, were associated with SNPs by generalized linear mixed models (Proc Glimmix) using JMP Genomics (SAS Institute, Cary, NC, USA). The genetic similarity matrix between individuals was first computed as identity by descent of each pair for the k matrix and used as a random effect. Using top principal components as covariates enabled correction for stratification in the population^72^,^73^. Additionally, genotype and gender were used as fixed effects, day of birth was used as a random effect, and piglet weight was used as a covariate. A genotype test for association of SNP alleles was also performed. To correct for multiple testing, a false discovery rate (FDR) of 5% was used.

### Replication in a second herd of cross-bred piglets

In total 794 cross-bred animals of the breeds German Landrace (DL) and Large White (DE) were used. The data were collected from research farm of the Institute of Animal Breeding and Husbandry of the University Kiel. The 1-min-backtests were repeatedly performed at an age of 12 and 19 days. The most significant SNPs in *PER1* (ASGA0055092) and *CTC1* (ASGA0105202) were used for genotyping by pyrosequencing. For association analysis generalized linear mixed models (Proc Glimmix; JMP Genomics (SAS Institute, Cary, NC, USA)) were used with the kindship matrixes of individuals considered as random effect, genotype and gender as fixed effects. To correct for multiple testing, a false discovery rate (FDR) was used.

### Quantitative PCR

We measured the levels of 37 hypothalamus transcripts (Supplementary Table S5) from 184 animals with tissue samples available using quantitative PCR (qPCR) with the Fluidigm BioMark HD System. The genes selected for analysis harbor SNPs represented on the Illumina PorcineSNP60 BeadChip that showed significant association with backtest traits. Samples for transcript abundance measurements were taken immediately after exsanguinations at about 180 day of age and snap frozen in liquid nitrogen until use. For qPCR, pre-amplification sample mixtures were prepared containing 2.7 μL of the diluted cDNA and primers (Supplementary Table S5), 3 μL SSO Evagreen supermix (BioRad), and 0.3 μL 20× sample-loading reagent. Separately, an assay mixture was prepared for each primer pair and this included 2.25 μL assay loading reagent, 0.25 μL 100 μM forward and reverse primer, and 2.5 μl 2× assay-loading reagent. The dynamic array was first primed with control line fluid and then loaded with the sample and assay mixtures via the appropriate inlets using an IFC controller. The array chip was placed in the BioMark Instrument for PCR at 95 °C for 10 min, followed by 30 cycles at 95 °C for 15 s and 60 °C for 1 min. Each sample was investigated in duplicate. The data were analyzed with real-time PCR analysis software in the BioMark HD instrument (Fluidigm Corporation, San Francisco, CA). Data were normalized using *HPRT1* and *RPS11* as internal controls.

### eQTL detection

The expression levels of 37 transcripts from the hypothalamus of 184 individuals representing a subset of those used for backtests were subjected to a mixed-model analysis of variance using JMP Genomics (Proc Mixed; SAS Institute, Rockville, MD). As in the GWAS of backtest traits, an IBD matrix between individuals was used as a random effect and top principal components as covariates corrected for stratification in the population. Additionally, genotype, gender, and the batch of RT-PCR were used as fixed effects, and weight at slaughter and average mean of housekeeping gene CT values were used as covariates. To correct for multiple testing, a false discovery rate (FDR) at 5% was used. We defined an eQTL as ‘cis’ if an associated SNP was located within an area less than 1 Mb from the gene. All other eQTL were considered as ‘trans’.

## Additional Information

**How to cite this article**: Ponsuksili, S. *et al.* Integrated Genome-wide association and hypothalamus eQTL studies indicate a link between the circadian rhythm-related gene *PER1* and coping behavior. *Sci. Rep.* **5**, 16264; doi: 10.1038/srep16264 (2015).

## Supplementary Material

Supplementary Table 1

Supplementary Table 2

Supplementary Table 3

Supplementary Table 4

Supplementary Table 5

Supplementary Table 6

## Figures and Tables

**Table 1:** Median, minimum (Min), and maximum (Max) of the backtest raw data for latency until the first response, duration of response and frequency of response in piglets (N = 294) at 5, 12, 19 and 26 days (d) of age.

Trait		Median	Min	Max
Latency (L) until the first response (in seconds)				
L at 5 days	(Ld5)	10.5	1	60
L at 12 days	(Ld12)	14	1	60
L at 19 days	(Ld19)	13	1	60
L at 26 days	(Ld26)	16	1	60
Duration (D) of all response (in seconds)				
D at 5 days	(Dd5)	19	0	53
D at 12 days	(Dd12)	17	0	47
D at 19 days	(Dd19)	18.5	0	49
D at 26 days	(Dd26)	15	0	48
Frequency (F) of response (number)				
F at 5 days	(Fd5)	3	0	8
F at 12 days	(Fd12)	2	0	5
F at 19 days	(Fd19)	2	0	7
F at 26 days	(Fd26)	2	0	6

**Table 2:** Markers significantly associated with backtest traits including latency (L) until the first response, duration (D) of responses and frequency (F) of responses in piglets at 5, 12, 19 and 26 days (d) of age.

SNP ID	Backtest	−log10	% variance	FDR	SSC (Mb)	Gene
ALGA0090009	Dd5	6.15	9.5	0.018	16 (31.0)	HCN1
ALGA0112274	Dd5	6.02	9.3	0.018		
ALGA0090013	Dd5	5.86	9.0	0.018	16 (31.0)	HCN1
DIAS0001557	Dd5	5.69	8.8	0.018	12 (55.9)	RANGRF
M1GA0008168	Dd5	5.50	8.5	0.018	5 (106.4)	
ALGA0067468	Dd12	5.63	8.7	0.035	13 (3.4)	
ALGA0067470	Dd12	5.63	8.7	0.035	13 (3.3)	
ASGA0102838	Dd12	5.50	8.5	0.035	12 (55.6)	
ASGA0104602	Dd12	5.49	8.5	0.035	2 (15.5)	
ASGA0055092	Dd12	5.35	8.3	0.035	12 (55.8)	PER1
ASGA0055092	Dd19	7.45	11.3	0.001	12 (55.8)	PER1
ASGA0102838	Dd19	7.38	11.2	0.001	12 (55.6)	
ALGA0066975	Dd19	6.59	10.1	0.003	12 (55.7)	
ALGA0121951	Dd19	6.57	10.1	0.003	12 (55.9)	
ALGA0095323	Dd19	6.34	9.7	0.004	17 (50.7)	
DIAS0001557	Dd26	6.23	9.6	0.019	12 (55.9)	RANGRF
ASGA0055076	Dd26	6.07	9.3	0.019	12 (55.2)	ATP1B2
DIAS0000983	Dd26	5.86	9.0	0.019	5 (7.3)	
ALGA0107834	Dd26	5.78	8.9	0.019	3 (139.8)	ASGR1
ASGA0085214	Dd26	5.55	8.6	0.019	12 (54.6)	
ALGA0115736	Fd5	6.33	9.9	0.021	2 (1.5)	liprin-alpha-1-like
ASGA0102974	Fd5	5.65	8.8	0.041		
ASGA0076061	Fd5	5.57	8.7	0.041	17 (32.2)	LOC100511790
CASI0009812	Fd5	5.36	8.4	0.045	4 (92.9)	
ASGA0040214	Fd5	5.23	8.2	0.045	8 (141.7)	
ALGA0056876	Fd12	6.60	8.9	0.007	10 (10.6)	
ASGA0102838	Fd12	6.54	10.1	0.007	12 (55.6)	
ASGA0055092	Fd12	6.21	9.6	0.010	12 (55.8)	PER1
ALGA0066975	Fd12	5.85	9.1	0.011	12 (55.9)	
ALGA0066986	Fd12	5.82	9.0	0.011	12 (56.1)	
ALGA0115736	Fd19	5.53	8.6	0.033	2 (1.5)	liprin-alpha-1-like
ASGA0102838	Fd19	5.47	8.6	0.033	12 (55.6)	
ASGA0055092	Fd19	5.46	8.5	0.033	12 (55.8)	PER1
H3GA0056700	Fd19	5.31	8.3	0.033		
ALGA0066975	Fd19	5.23	8.2	0.033	12 (55.9)	
ALGA0096195	Fd26	5.19	8.1	0.043	17 (64.1)	
ALGA0104320	Fd26	5.11	8.0	0.043	1 (22.5)	
ALGA0107609	Fd26	5.11	8.0	0.043	1 (22.3)	
ASGA0094670	Fd26	5.11	8.0	0.043	1 (22.5)	
ASGA0055092	Fd26	5.10	8.0	0.043	12 (55.8)	PER1
ALGA0066975	Ld12	6.47	9.9	0.006	12 (55.9)	
ASGA0055092	Ld12	6.39	9.8	0.006	12 (55.8)	PER1
ALGA0121951	Ld12	6.34	9.7	0.006	12 (55.9)	
ALGA0066986	Ld12	6.12	9.4	0.006	12 (56.1)	
ASGA0099599	Ld12	6.12	9.4	0.006	12 (55.1)	

**Table 3:** Markers significantly associated with backtest traits of total latency until the first response (tL), total duration of all responses (tD), total frequency of all responses (tF) and backtest type in piglets.

SNP_ID	Trait	−log 10	% variance	FDR	SSC (Mb)	Gene
DIAS0001557	tL	6.79	10.4	0.0073	12 (56.0)	RANGRF
ASGA0099599	tL	6.10	9.4	0.0101	12 (55.1)	
H3GA0034753	tL	5.96	9.2	0.0101	12 (56.1)	LOC100739126
ALGA0066960	tL	5.83	9.0	0.0101	12 (55.2)	WRAP53
M1GA0016958	tL	5.83	9.0	0.0101	12 (55.2)	WRAP53
ALGA0066969	tL	5.83	9.0	0.0101	12 (55.6)	
ALGA0107834	tL	5.81	8.9	0.0101	3 (139.8)	ASGR1
ASGA0055092	tL	5.70	8.8	0.0111	12 (55.8)	PER1
ASGA0102838	tL	5.57	8.6	0.0111	12 (55.6)	
ALGA0027884	tL	5.56	8.6	0.0111		
ASGA0055092	tF	8.36	13.3	0.0002	12 (55.8)	PER1
ALGA0066975	tF	7.99	12.8	0.0002	12 (55.9)	
ALGA0121951	tF	7.84	12.6	0.0002	12 (55.9)	
H3GA0034753	tF	7.79	12.5	0.0002	12 (56.1)	LOC100739126
ASGA0102838	tF	7.57	12.2	0.0002	12 (55.6)	
ALGA0115736	tF	7.57	12.1	0.0002	2 (1.5)	liprin-alpha-1-like
ASGA0105202	tF	7.48	12.0	0.0002	12 (55.9)	CTC1
MARC0073387	tF	7.48	12.0	0.0002	12 (55.9)	
M1GA0016964	tF	7.33	11.8	0.0002	12 (55.8)	
ALGA0066960	tF	7.16	11.5	0.0003	12 (55.2)	WRAP53
ASGA0055092	tD	6.84	10.5	0.0039	12 (55.8)	PER1
ASGA0102838	tD	6.58	10.1	0.0039	12 (55.6)	
ALGA0096195	tD	6.38	9.8	0.0039	17 (64.1)	
ALGA0066975	tD	6.27	9.6	0.0039	12 (55.9)	
MARC0035809	tD	6.25	9.6	0.0039	10 (72.9)	
ALGA0066986	tD	6.25	9.6	0.0039	12 (56.1)	
ALGA0107834	tD	6.24	9.6	0.0039	3 (139.8)	ASGR1
ASGA0049082	tD	6.10	9.4	0.0044	10 (73.9)	
ALGA0060087	tD	6.07	9.3	0.0044	10 (73.6)	
ALGA0112457	tD	6.03	9.3	0.0044	6 (31.8)	LONP2
ALGA0107834	coping type	5.72	8.8	0.0546	3 (139.8)	ASGR1
ALGA0095323	coping type	5.63	8.6	0.0546	17 (50.7)	
MARC0035809	coping type	5.34	8.2	0.0712	10 (72.9)	
DIAS0001557	coping type	5.11	7.9	0.0807	12 (56.0)	RANGRF
ALGA0112457	coping type	5.03	7.7	0.0807	6 (31.8)	LONP2
ALGA0096195	coping type	4.95	7.6	0.0807	17 (64.1)	
ASGA0055092	coping type	4.90	7.6	0.0807	12 (55.8)	PER1
MARC0062446	coping type	4.76	7.4	0.0807	18 (2.9)	
ASGA0076061	coping type	4.65	7.2	0.0807	17 (32.2)	C17H20orf26
ASGA0102838	coping type	4.62	7.2	0.0807	12 (55.6)	

**Table 4:** Markers which significantly associated with backtest traits were used for association study in a second herd for validation; latency (L), duration (D) and frequency (F) of responses in piglets were recorded at 12 and 19 days (d) of age as well as the totalizing data of both ages (tD, tL and tF)

Trait	Sample Size	*p-vaule*	FDR	SNP	Gene
Dd12	794	0.185	0.185	ASGA0105202	CTC1
Dd19	791	0.583	0.583		
tD	791	0.190	0.190		
Fd12	794	0.017	0.017		
Fd19	794	0.718	0.718		
tF	794	0.493	0.493		
Ld12	794	0.346	0.346		
Ld19	791	0.849	0.849		
tL	791	0.657	0.657		
Dd12	788	0.015	0.030	ASGA0055092	PER1
Dd19	785	0.003	0.006		
tD	785	0.001	0.003		
Fd12	788	0.006	0.011		
Fd19	788	0.191	0.381		
tF	788	0.009	0.017		
Ld12	788	0.007	0.015		
Ld19	785	0.035	0.069		
tL	785	0.013	0.026		
